# Histopathologic Evaluation of Nonalcoholic Fatty Liver Disease in Hypothyroidism-Induced Rats

**DOI:** 10.1155/2016/5083746

**Published:** 2016-04-07

**Authors:** Şule Demir, Mustafa Ünübol, Serap Ünübol Aypak, Emrah İpek, Serdar Aktaş, Gamze Sevri Ekren, Murat Yılmaz, Recai Tunca, Engin Güney

**Affiliations:** ^1^Department of Internal Medicine, Adnan Menderes University Faculty of Medicine, 09100 Aydin, Turkey; ^2^Division of Endocrinology, Department of Internal Medicine, Adnan Menderes University Faculty of Medicine, 09100 Aydın, Turkey; ^3^Department of Biochemistry, Adnan Menderes University Faculty of Veterinary Medicine, 09100 Aydın, Turkey; ^4^Department of Pathology, Adnan Menderes University Faculty of Veterinary Medicine, 09100 Aydın, Turkey; ^5^Department of Pharmacology Toxicology, Adnan Menderes University Faculty of Veterinary Medicine, 09100 Aydın, Turkey; ^6^Department of General Surgery, Adnan Menderes University Faculty of Medicine, 09100 Aydın, Turkey

## Abstract

It is speculated that thyroid hormones may be involved in nonalcoholic fatty liver disease (NAFLD) pathogenesis. A literature scan, however, demonstrated conflicting results from studies investigating the relationship between hypothyroidism and NAFLD. Therefore, our study aims to evaluate NAFLD, from the histopathologic perspective, in hypothyroidism-induced rats. Wistar rats were divided into 2 groups: the experimental group consumed water containing methimazole 0.025% (MMI, Sigma, USA) for 12 weeks and the control group consumed tap water. At the end of week 12, serum glucose, ALT, AST, triglyceride, HDL, LDL, TSH, fT4, fT3, visfatin, and insulin assays were performed. Sections were stained with hematoxylin-eosin and “Oil Red-O” for histopathologic examination of the livers. In our study, we detected mild hepatosteatosis in all hypothyroidism-induced rats. There was statistically significant difference with respect to obesity between the two groups (*p* < 0.001). The mean fasting blood glucose was 126.25 ± 23.4 mg/dL in hypothyroidism-induced group and 102.63 ± 15.51 mg/dL in the control group, with a statistically significant difference between the groups (*p* = 0.032). The two groups did not differ statistically significantly with respect to visfatin levels (*p* > 0.05). In conclusion, we found that hypothyroidism-induced rats had mild hepatosteatosis as opposed to the control group histopathologically. Our study indicates that hypothyroidism can cause NAFLD.

## 1. Introduction

Thyroid hormones have important roles in body weight, adipogenesis, energy homeostasis, and regulation of carbohydrate and lipid metabolism [1, 2]. Clinical studies of hypothyroidism have shown a possible association with metabolic syndrome, obesity, and impaired lipid metabolism [3, 4]. Nonalcoholic fatty liver disease (NAFLD) is a chronic hepatic disease, with a broad histopathologic spectrum ranging from a simple steatosis to nonalcoholic steatohepatitis (NASH), fibrosis, and cirrhosis [5, 6]. Closely associated with insulin resistance, hypertension, dyslipidemia, and obesity, NAFLD is believed to reflect the hepatic component of the metabolic syndrome [7].

It is speculated that thyroid hormones may be involved in NAFLD pathogenesis [8]. A literature scan, however, demonstrated conflicting results from studies investigating the relationship between hypothyroidism and NAFLD [9–13]. Chung et al. [13] reported that ultrasonographically determined NAFLD development was more frequent among patients with hypothyroidism compared to healthy controls. However, Ittermann et al. [12] identified no relationship between hepatosteatosis and hypothyroidism. In their article reviewing the studies on the relationship between thyroid dysfunction and NASH/NAFLD, Eshraghian and Jahromi [8] emphasized that subclinical hypothyroidism and hypothyroidism were more common in patients with NASH/NAFLD and that hypothyroidism was an independent risk factor for NASH/NAFLD in some studies, although some recent studies have reported no correlation. Hence, the need for new studies to elucidate the relationship between NAFLD and hypothyroidism was highlighted.

Therefore, our study aims to evaluate NAFLD, from the histopathologic perspective, in hypothyroidism-induced rats.

## 2. Materials and Methods

### 2.1. Ethics Board Approval

For the present study, ethics board approval of the Local Ethics Committee for Animal Experiments of Adnan Menderes University (ADÜ-HADYEK) was received on 27/01/2015 (decision number 64583101/2015/002).

### 2.2. Study Protocol

Sixteen adult male Wistar Albino rats (8–12 weeks, 250 g–330 g) were used for this study. The rats were maintained at room temperature at 24°C ± 1, alternately for 12 hours under lit (07:00–19:00) and 12 hours at dark (19:00–07:00) conditions. All rats were monitored in the same environment and were fed with the same standard feed and were provided with water and feed* ad libitum*.

*Group 1 (Healthy Control Group (n* = 8*))*. The rats in this group were only given daily drinking water and feed.

*Group 2 (Hypothyroidism-Induced Group (n* = 8*))*. Hypothyroidism was induced by adding methimazole 0.025% (MMI, Sigma, USA) to drinking water for 12 weeks [14].

Rats' bodyweights were measured weekly. At the end of week 12, bodyweights following a 12-hour fasting and nasoanal length were measured in the rats in all groups. Lee index was used to evaluate obesity. The index was calculated as follows: body weight (g) cubic root × 10/nasoanal length (mm). Results ≤0.300 were considered normal and results >0.300 were considered as obesity [15]. For anesthesia, intraperitoneal 40 mg/kg ketamine + 4 mg/kg xylazine was administered. Under anesthesia, blood was collected from systemic circulation and cervical dislocation was performed. The material for biochemical analysis was foil-wrapped and was transferred within a very short time to the biochemistry laboratory.

### 2.3. Serum and Blood Sample Preparation

Blood samples taken from all rats were collected in gel-containing serum tubes. Serum samples were divided into small portions in amounts sufficient for intended analyses and were kept at −20°C pending analyses (except glucose and ALT analyses, which were performed immediately). Serum glucose, ALT, AST, triglyceride, HDL, LDL, and insulin assays were performed using the commercial kits of BIOLABO (Maizy, France). Immunoassay based on specific sandwich Elisa method was used to determine free triiodothyronine (fT3) (SunRed, Biotechnology Company, Shanghai, Cat. number 201-11-0738), free thyroxine (fT4) (SunRed, Biotechnology Company, Shanghai, Cat. number 201-11-0736),* thyroid-stimulating hormone* (TSH) (SunRed, Biotechnology Company, Shanghai, Cat. number 201-11-0181), and visfatin (SunRed, Biotechnology Company, Shanghai, Cat. number 201-11-0472) levels in sera obtained from the rats. TSH kits had a measuring range of 0.03–6 mIU/L, sensitivity of 0.021 mIU/L, Intra-Assay CV of <10%, and Inter-Assay CV of <12%. sT4 kits had a measuring range of 0.5–150 pmol/L and sensitivity of 0.466 pmol/L.

### 2.4. Histopathologic Assessment

Livers were removed following euthanasia and were first washed with physiological saline solution. Recommendations of Ruehl-Fehlert et al. [16] were followed in trimming the livers and determining the sections to be examined. After washing, the liver was trimmed to include the left lateral lobe, caudate lob, and right and left medial lobe. The trimmed tissues were conveniently placed into cassettes and fixed in 10% neutral buffered formalin (Sigma-Aldrich) for 24–48 hours, after which the tissues were washed with running tap water for 6–8 hours and were then embedded in paraffin using the automatic tissue processing instrument (Leica TP1020). Sections of paraffin-embedded tissues, 5 *μ*m thick, were transferred onto slides (Thermo Scientific). Sections were stained with hematoxylin-eosin for histopathologic examination. To demonstrate fat vacuoles in the liver, trimmed tissues were sliced in 10 *μ*m thickness with a freezing microtome. The sections were then stained using the “Oil Red-O” method. The obtained liver preparations were examined under light microscope (Olympus BX51) and microscopic digital photographs were taken (Olympus C-5050), which were transferred into computer.

### 2.5. Statistical Analysis

Data obtained from the study were analyzed using the SPSS 17.0 (Statistical Package for the Social Science, version 17.0). For comparative analyses, distribution pattern was first determined using the Kolmogorov-Smirnov test and then Student's *t*-test and chi-square were used. *p* values <0.05 were considered statistically significant.

## 3. Results

### 3.1. Obesity Assessment

Mean fasting bodyweight measured on the first day of the study was 287.25 ± 15.78 g in the hypothyroidism group and 282.88 ± 33.59 g in the control group, which were similar (*p* > 0.05). Mean fasting bodyweight measured on the last day of the study was 297.38 ± 19.62 g in the hypothyroidism group and compared to 290.75 ± 33.34 g in the control group, with no statistically significant difference (*p* > 0.05). With obesity assessment based on Lee index, baseline measurements were comparable between the groups (*p* > 0.05), while Lee indices at the end of the study were 0.309 ± 0.005 in the hypothyroidism compared versus 0.292 ± 0.008 in the control group, with a statistically significant difference between the two groups (*p* < 0.001). At baseline, rats in both groups were not obese. At the end of the study, all rats in hypothyroidism group developed obesity. Obesity was noted in only one rat in the control group. There was statistically significant difference with respect to obesity between the two groups (*p* < 0.001).

### 3.2. Evaluation of Biochemical Parameters

TSH in the hypothyroidism group was 2.16 ± 0.35 mIU/L and differed statistically significantly from the control group (*p* < 0.001). fT4 in the hypothyroidism group was 10.33 ± 4.46 pmol/L, which was statistically significantly lower compared to the control group (*p* = 0.006). Similarly, fT3 in the hypothyroidism group was 5.32 ± 0.62 nmol/L and was statistically significantly lower compared to the control group (*p* < 0.001). The two groups did not differ statistically significantly with respect to visfatin levels (*p* > 0.05). All biochemical parameters are provided in Table 1.

### 3.3. Pathology Results

#### 3.3.1. Macroscopic Results

In all hypothyroidism-induced rats, livers appeared pale and brittle. No macroscopic change was observed in the livers of the rats in the control group.

#### 3.3.2. Microscopic Histopathologic Results

**Figure 1:**
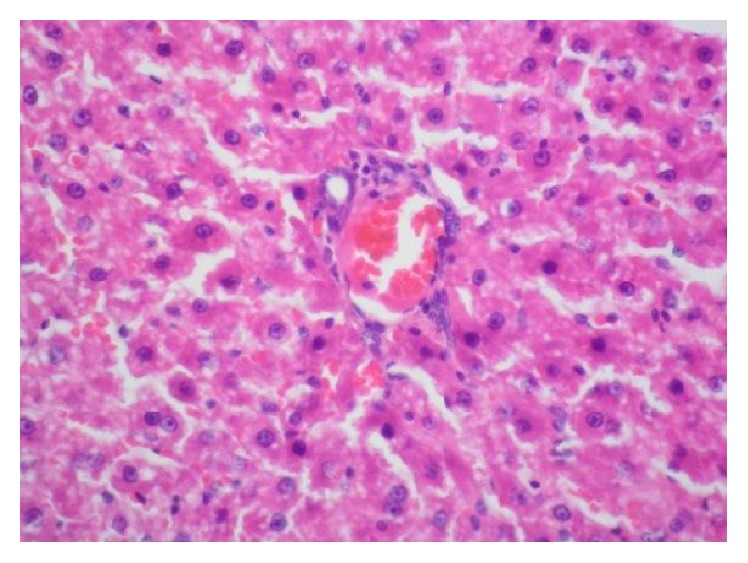
With HE staining, clear microvesicular vacuoles with sharp edges affecting hepatocyte to varying degrees and distributed randomly were seen.

**Figure 2:**
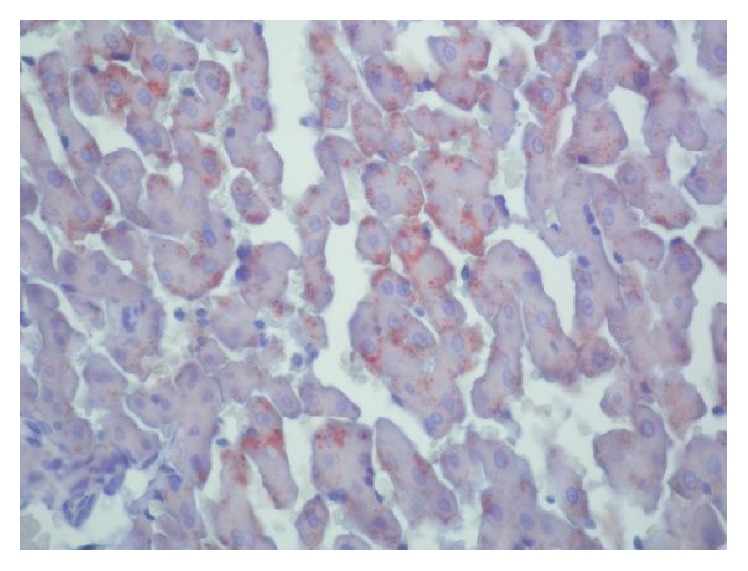
The vacuoles were determined to be fat vacuoles with Oil Red staining.

Livers of all hypothyroidism-induced rats involved mild steatosis. No microscopic steatosis was observed in the livers of the rats in the control group. There was statistically significant difference with respect to microscopic hepatosteatosis between the two groups (*p* < 0.001). With HE staining, in livers of all hypothyroidism-induced rats, clear microvesicular vacuoles with sharp edges affecting hepatocyte to varying degrees and distributed randomly were seen (Figure 1). The vacuoles were determined to be fat vacuoles with Oil Red staining (Figure 2). Nuclei of hepatocytes were of normal appearance and location. However, impaired structure of remark cords and mild parenchymal degeneration were the other observed changes.

With Oil Red staining in the livers of the rats in the control group, there were hardly remarkable, randomly distributed, sporadic microvesicular fat vacuoles. Both the hypothyroidism group and the control group had portally located mild mononuclear cell infiltrations consisting of lymphocytes and macrophages.

## 4. Discussion

In our study, we detected mild hepatosteatosis in all hypothyroidism-induced rats. Randomly distributed sporadic fat vacuoles observed in the livers of rats in the healthy control group can normally be found in rat livers [17]. The number of affected hepatocytes and the severity of fat vacuoles build-up in the livers of hypothyroidism-induced rats indicate that hypothyroidism leads to fatty changes in the liver. Portally located mild mononuclear cell accumulations in both hypothyroidism-induced and control groups are changes with unknown relevance that can be seen in rat livers [17].

A study by Liangpunsakul and Chalasani [18] included 174 patients with NASH diagnosed with liver biopsy. Hypothyroidism was detected in 15% of the patients with NASH and in 7.2% of the control group. The authors suggested that hypothyroidism was more prevalent among NASH patients compared to the control group. In a study by Silveira et al. [19], frequency of thyroid dysfunction was 13% in patients with primary biliary cirrhosis, 11% in patients with primary sclerosing cholangitis, and 25% in patients with NAFLD and 20% of NAFLD patients had hypothyroidism. In the sectional study by Pagadala et al. [20], 21% of the patients were diagnosed with NASH/NAFLD with liver biopsy and 25% of non-NASH patients were diagnosed with NAFLD and 12.8% of the healthy control group had hypothyroidism. The authors reported a higher prevalence of hypothyroidism in NAFLD patients compared to the control group. In a study by Parikh et al. [21] in Western India, 16.8% of the patients with USG-diagnosed NAFLD had hypothyroidism, which was significantly higher compared to the control group. Hypothyroidism was underlined as a risk factor independent from the known risk factors for NAFLD. Mazo et al. [22] found the prevalence of hypothyroidism in NAFLD patients as 15.5% compared to 15.7% in NASH patients and 15.2% in patients with steatosis. In the study by Carulli et al. [10] liver biopsies demonstrated NAFLD in 69 patients, MASH in 44 patients, and steatosis in 25 patients. Patients with NASH had significantly higher TSH levels compared to patients with steatosis, and the authors emphasized that TSH level can be an independent positive risk factor for NASH. Xu et al. [23] monitored 63 patients with subclinical hypothyroidism and 35 euthyroid healthy control groups for a median of 4.92 years for NAFLD development. The incidence of NAFLD development in patients with subclinical hypothyroidism was significantly higher than in euthyroid individuals.

However, unlike these studies, there are studies demonstrating a lack of relationship between hypothyroidism and NASH/NAFLD. In a study by Ittermann et al. [12], no relationship was found between hepatosteatosis and hypothyroidism. Hepatosteatosis was not related with TSH or fT3 concentrations but there was a relationship with low fT4 concentrations. A study by Eshraghian et al. [11] did not determine a correlation between NAFLD and thyroid dysfunction. TSH, fT4, and fT3 were not statistically different in patients with and without NAFLD.

Our literature scan did not yield a study evaluating hepatosteatosis, NAFLD development with liver biopsy in patients with hypothyroidism. In our study, we histopathologically evaluated if hypothyroidism leads to hepatosteatosis, NAFLD development. Because liver biopsy would be an invasive procedure to be used in hypothyroidism patients, our study used an animal model. We histopathologically determined with this study that hypothyroidism leads to mild hepatosteatosis in rats.

We believe that many factors are involved in explaining the mechanism of the relationship between hypothyroidism and NAFLD. As one of these factors, obesity is commonly seen together with NAFLD. In morbidly obese patients, NAFLD frequency is as high as 90% and advanced disease (e.g., NASH) is seen in 9 to 40%. The relationship between BMI and the extent of fat build-up and hepatic damage has been shown in a number of studies [24–26]. In obese cohorts scheduled to receive bariatric surgery, it was reported that steatohepatitis prevalence could be from 37% to as high as 91% with biopsy [27].

Studies supporting the relationship between hypothyroidism and NAFLD emphasize that this relationship may be associated with obesity development secondary to hypothyroidism [28, 29].

Eshraghian et al. [11] found a relationship between central obesity and NAFLD but not between hypothyroidism and NAFLD. In our study, all hypothyroidism-induced rats developed obesity according to the Lee index. Obesity was observed in only one rat in the healthy control group. There was a statistically significant difference between the two groups with respect to obesity frequency. We believe that obesity is one of the major determinants in the relationship between hypothyroidism and NAFLD.

Thyroid hormones mediate the effects on lipid metabolism via thyroid hormone receptor *β* expressed from the liver [30]. Hypothyroidism can also lead to increased serum triglyceride levels by decreasing hepatic lipoprotein lipase activity [31]. Several studies demonstrated increased LDL cholesterol levels in subclinical hypothyroidism patients [4, 32–34].

Fabbrini et al. [35] found higher plasma VLDL and triglyceride concentrations in subclinical hypothyroidism patients compared to euthyroid individuals. Several studies demonstrated a negative correlation between triglyceride levels and fT4 levels [36, 37]. Hypertriglyceridemia is known as a factor that contributes to NAFLD development [38, 39]. Lipid profiles of patients diagnosed with NAFLD/NASH involve significant increase in LDL cholesterol and triglyceride levels [40]. In an animal study by Cable et al. [41], in which a hepatosteatosis model was induced, the animals were given liver-targeted thyroid hormone receptor agonist. The study demonstrated that treatment with liver-targeted thyroid hormone receptor agonist in animals with coexisting NASH and hyperlipidemia decreased LDL cholesterol and could provide additional therapeutic benefits.

In our study, the LDL level of hypothyroidism-induced group was statistically significantly higher compared to the healthy control group. The groups were not different with regard to serum triglyceride and HDL levels. We believe that this change in the lipid profile supports NAFLD development in the hypothyroidism-induced group.

Most NAFLD patients have insulin resistance and diabetes mellitus usually accompanies this [42]. Liangpunsakul and Chalasani [18] determined that 38.5% of the patients with NAFLD had diabetes mellitus. Pagadala et al. [20] found diabetes mellitus in 42.7% of the patients with NAFLD, which was significantly higher compared to the control group. It has been reported that hypothyroidism may be associated with insulin resistance, which may be decreased if hypothyroidism is treated [43]. Smithson [44] determined that hypothyroidism was more common in diabetic patients compared to overall population. Gronich et al. [45] concluded that hypothyroidism was a risk factor for diabetes mellitus. The authors emphasized that the risk of diabetes mellitus could be reduced by identifying and treating hypothyroidism. In our study, mean fasting blood glucose was 126.25 ± 23.4 mg/dL in hypothyroidism-induced group and 102.63 ± 15.51 mg/dL in the control group, with a statistically significant difference between the groups (*p* = 0.032). Our results supported that hypothyroidism increases the risk of diabetes mellitus. Pronounced hyperglycemia in the hypothyroidism-induced group was considered as one of the consequences that support NAFLD development.

The role of adipokines in NAFLD pathogenesis was demonstrated by previous studies [46, 47]. Visfatin, an adipokine, is a hormone, plasma levels of which are also associated with obesity, visceral fat, type 2 diabetes, and metabolic syndrome [48]. High levels of visfatin were found in obesity, type 2 diabetes, and metabolic syndrome [49, 50]. There is limited data on the relationship between NAFLD and visfatin levels. Lower visfatin levels were observed in patients with NASH compared to those with NAFLD [47, 51]. Several studies attempted to describe the mechanism between hypothyroidism and NAFLD through adipokines. Some of these studies identified no relationship between adipokines and hypothyroidism [52, 53]. Contradicting views exist on the relationship between visfatin and hypothyroidism. One study found a positive correlation between TSH and visfatin levels and negative correlation between fT3, fT4, and visfatin levels [54]. Another study observed higher visfatin levels in both subjects with hyperthyroidism and those with hypothyroidism compared to controls and found increased visfatin levels following treatment [55]. There are studies with contradicting results on the role of thyroid hormones on visfatin regulation [56, 57]. Ozkaya et al. [54] argued that the effect of hyperthyroidism on several metabolic parameters could be mediated by visfatin although the effect of thyroid dysfunction on the production and release of adipocytokines has not been fully understood. In our study, the two groups did not differ in visfatin levels. Our study supports the view that visfatin is not involved in the development of NAFLD secondary to hypothyroidism.

## 5. Conclusion

We found that hypothyroidism-induced rats had mild hepatosteatosis as opposed to the control group histopathologically. Our study indicates that hypothyroidism can cause NAFLD.

## Figures and Tables

**Table 1:** Results of biochemical parameters.

	Hypothyroidism (*n* = 8)	Controls (*n* = 8)	*p* ^*∗*^
TSH (mIU/L)	2.16 ± 0.35	0.84 ± 0.06	**<0.001**
fT4 (pmol/L)	10.33 ± 4.46	16.07 ± 2.19	**0.006**
fT3 (nmol/L)	5.32 ± 0.62	7.47 ± 0.12	**<0.001**
Visfatin (ng/mL)	88.04 ± 7.29	89.67 ± 5.92	>0.05
Fasting plasma glucose (mg/dL)	126.25 ± 23.4	102.63 ± 15.51	**0.032**
ALT (U/L)	58.38 ± 22.9	38.75 ± 6.04	**0.034**
AST (U/L)	153.63 ± 35.1	98.5 ± 17.65	**0.001**
Triglyceride (mg/dL)	38.5 ± 6.89	37.63 ± 11.36	>0.05
HDL (mg/dL)	24.5 ± 3.34	23 ± 1.93	>0.05
LDL (mg/dL)	53 ± 10.85	35.88 ± 3.8	**0.001**
Fasting insulin levels (mU/mL)	1.19 ± 0.5	1 ± 0.36	>0.05

^**∗**^Student's *t*-test was used.
